# Advances in promoter engineering strategies for enhanced recombinant protein expression in plants

**DOI:** 10.3389/fpls.2025.1747353

**Published:** 2026-01-19

**Authors:** Shekoofeh Sadravi, June Lee, Jianfeng Xu

**Affiliations:** 1 Arkansas Biosciences Institute, Arkansas State University, Jonesboro, AR, United States; 2 Department of Biological Sciences, Arkansas State University, Jonesboro, AR, United States; 3 College of Agriculture, Arkansas State University, Jonesboro, AR, United States

**Keywords:** molecular farming, promoter engineering, recombinant protein expression, synthetic biology, transgene regulation

## Abstract

Plant-based expression systems, known as molecular farming, have emerged as sustainable and scalable platforms for producing recombinant proteins used in pharmaceuticals, industrial enzymes, and agricultural products. Among the key determinants of transgene performance, promoter elements play a central role in defining transcriptional strength, specificity, and regulation. This review highlights current advances in promoter engineering tailored for plant systems, encompassing natural, synthetic, hybrid, inducible, and tissue-specific promoters used in stable transgenic plants, transient expression systems, and plant cell cultures. The structural and functional features of promoter elements are discussed, along with strategies to mitigate challenges such as transcriptional silencing, genomic context dependency, and variability cross species and production platform. Emerging synthetic biology tools, such as CRISPR-based transcriptional control, high-throughput screening, and machine learning–assisted promoter design, are enabling the creation of tunable, orthogonal promoters suited for complex multigene expression. As promoter engineering continues to evolve, it remains foundational to advancing plant molecular farming and expanding the role of plants as versatile biofactories for high-value recombinant proteins.

## 1 Introduction

Recombinant protein production is a cornerstone of modern biotechnology, supporting applications in pharmaceutical manufacturing, industrial enzyme production, and agricultural innovation. Although bacterial and mammalian expression systems have long dominated the field, plant-based expression platforms, often referred to as molecular farming, have gained increasing attention as competitive alternatives (Eidenberger et al., 2023; Buyel, 2024). These systems offer several advantages, including low production costs, scalability, freedom from animal pathogens, and the capacity to perform complex post-translational modifications (Benvenuto et al., 2023; Buyel, 2024). Among plant-based systems, both whole plants (through stable transformation or transient expression) and plant cell cultures (such as tobacco BY-2 cells, rice suspension cultures, and moss bioreactors) have been successfully explored for producing therapeutic proteins, vaccines, and bioactive enzymes (Xu et al., 2016; Fischer and Buyel, 2020; Schillberg and Finnern, 2021).

Despite the promise of plant-based systems, achieving high and consistent recombinant protein yields remains a critical bottleneck (Eidenberger et al., 2023; Szarzanowicz et al., 2024; Xu et al., 2025). Expression efficiency is influenced by multiple factors, such as codon usage, mRNA stability, protein targeting and folding, and post-translational modifications (Obembe et al., 2011; Schillberg et al., 2019). However, the initial step of gene expression, transcriptional activation, is often identified as a major limiting factor. The level of mRNA accumulation, and ultimately the amount of protein produced, is critically determined by the strength, specificity, and regulation of the promoter driving the gene of interest (Hernandez-Garcia and Finer, 2014).

To overcome these limitations, promoter engineering has emerged as a key strategy, enabling enhanced transgene expression through optimization of core promoter elements, incorporation of enhancer sequences, and integration of synthetic regulatory motifs (Venter, 2007; Liu and Stewart, 2016). Moreover, precise control of gene expression in terms of timing, tissue specificity, and expression levels has become achievable through promoter engineering combined with complementary molecular tools such as transcription enhancers, insulators, and synthetic transcription factors (Koo et al., 2025).

Given these advances, promoter engineering has emerged as a pivotal strategy for enhancing recombinant protein production in plant molecular farming. Rather than relying solely on a small set of broadly active promoters, current approaches emphasize rational design, modularity, and platform-specific optimization. This review synthesizes recent advances in natural, synthetic, inducible, and tissue-specific promoter engineering, with particular emphasis on their functional performance across whole plants, transient expression systems, and plant cell cultures. By integrating mechanistic insights with practical constraints, we aim to provide a framework for selecting and designing promoters suited to diverse molecular farming applications.

## 2 Promoter architecture and functional elements

**Figure 1:**
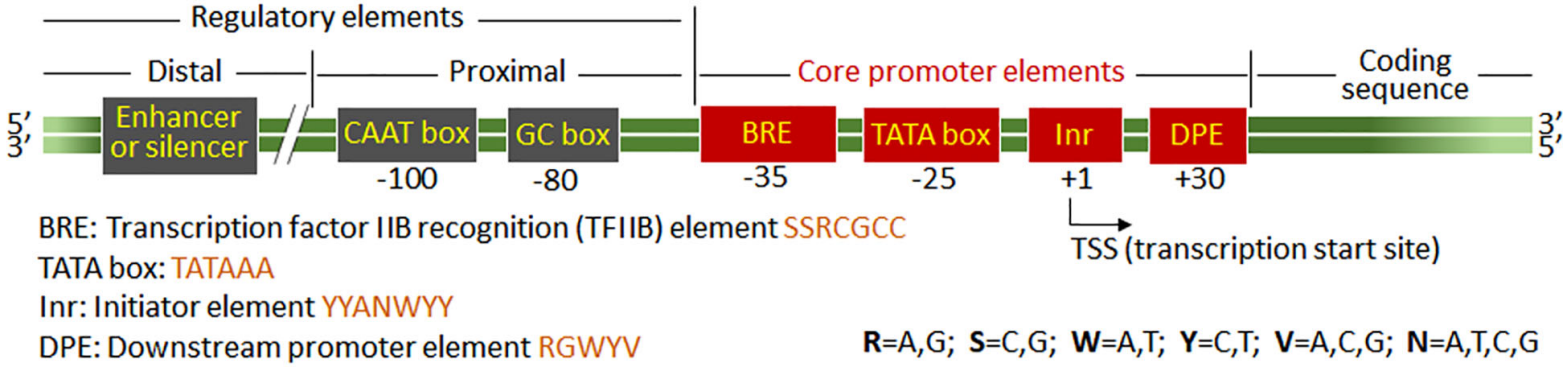
Classification of plant promoters by origin/design and regulatory behavior.

Promoters are DNA sequences that initiate gene transcription by providing binding sites for RNA polymerase and transcription factors (TFs), thereby controlling when, where, and to what extent genes are expressed (Venter, 2007; Hernandez-Garcia and Finer, 2014). Based on their *origin and design*, promoters can be broadly classified into two main categories (**Figure 1**): (1) natural promoters, derived from native organisms and retaining inherent regulatory features; (2) synthetic promoters, engineered from defined sequence motifs or integrating components from both natural and synthetic sources (hybrid promoters) to achieve customized expression patterns (Dey et al., 2015; Liu and Stewart, 2016). Regardless of their origin, promoters can be further categorized by their *regulatory behavior* into three major types (**Figure 1**): (a) Constitutive promoters, driving continuous expression; (b) Spatiotemporal promoters, conferring tissue- or stage-specific expression; and (c) Inducible promoters, enabling controlled expression in response to chemical or physical stimuli (Hernandez-Garcia and Finer, 2014; Ali and Kim, 2019). This framework facilitates the evaluation and rational design of gene expression systems for diverse applications.

**Figure 2:**
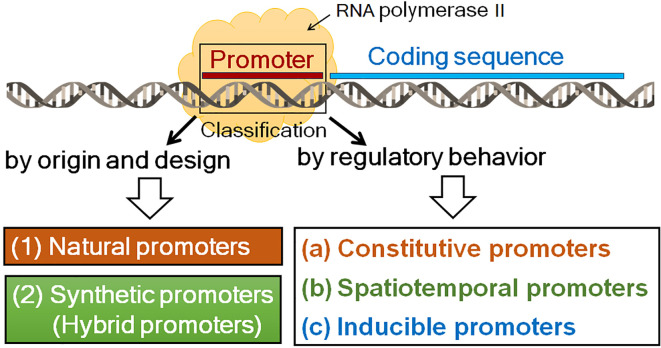
Schematic representation of promoter modular architecture illustrating core and distal elements. Not all the depicted elements are universal.

Central to promoter function is the core promoter (**Figure 2**), a short region located approximately −40 to +40 bp relative to the transcription start site (TSS) that serves as the assembly platform for the transcriptional machinery (Bulger and Groudine, 2011; Liu and Stewart, 2016). Key core promoter elements include the TATA box, a well-characterized motif that facilitates the precise positioning of RNA polymerase II; the Initiator (Inr) element, which overlaps the TSS and directs accurate transcription initiation; the TFIIB recognition elements (BRE) located upstream or downstream of the TATA box; and the downstream promoter element (DPE), which functions in TATA-less promoters (Parry et al., 2010; Hernandez-Garcia and Finer, 2014). The composition and arrangement of these elements influence transcription efficiency, promoter strength, and regulatory responsiveness (Kadonaga, 2012). Gene expression is further refined by additional cis-regulatory elements (**Figure 2**). Proximal elements, typically within 100–200 bp upstream of the core promoter, include TF-binding motifs such as the CAAT box and GC-rich regions that modulate transcription initiation (Liu and Stewart, 2016). More distant regulation is mediated by distal elements, including enhancers, silencers, and insulators, which can act over long genomic distances through chromatin looping to enable complex spatiotemporal and environmental control of gene expression (Bulger and Groudine, 2011; Spitz and Furlong, 2012). Together, these modular and hierarchical elements define promoter architecture in both natural gene regulation and synthetic promoter design for biotechnological applications.

## 3 Natural and synthetic promoters in plant molecular farming

### 3.1 Natural promoters and their limitations

Natural promoters have long served as the foundation for driving transgene expression in plant biotechnology. These promoters are typically chosen for their well-characterized regulatory features, broad activity across plant species, and compatibility with existing cloning systems (Hernandez-Garcia and Finer, 2014). Some of the most widely used natural promoters include the cauliflower mosaic virus 35S promoter (CaMV35S) (Knodler et al., 2023), the maize ubiquitin-1 promoter (ZmUbi-1) (Christensen et al., 1992), the rice actin-1 promoter (OsAct-1) (McElroy et al., 1990), and the *Arabidopsis* ubiquitin promoter-10 (AtUbi-10) (Norris et al., 1993; Grefen et al., 2010). Their relative strengths, regulatory modes, and key limitations are summarized in **Table 1**.

**Table 1:** Common natural promoters used in recombinant protein expressions in plants.

Promoter	Species source	Relative strength	Regulation type	Limitations
CaMV35S	*Cauliflower mosaic virus*	High (in dicots)	Constitutive (broad range)	Reduced activity in monocots; may be silenced in stable lines; not inducible
Ubiquitin (Ubi) (e.g., AtUbi-10, ZmUbi-1)	*Arabidopsis thaliana*, *Zea mays*	Moderate to high	Constitutive	Can have variable expression depending on species and tissue
Actin (Act); (e.g., OsAct-1)	*Oryza sativa*	High (in monocots)	Constitutive (strong in rapidly dividing tissues)	Less characterized in dicots; potential developmental regulation
Nopaline synthase (Nos)	*Agrobacterium tumefaciens*	Low to moderate	Constitutive	Generally weaker than CaMV35S; less reliable in monocots
Rubisco small subunit (RbcS)	Various plant species	Moderate to high	Green tissues (light-inducible)	Limited to photosynthetic tissues; not ideal for non-green organs
Elongation factor 1α (EF1α)	*A. thaliana*, *O. sativa*, others	High	Constitutive	Activity can be variable depending on developmental stage

The CaMV35S promoter is one of the most commonly used constitutive promoters in plant systems due to its strong transcriptional activity in many dicotyledonous species (Benfey and Chua, 1990; Venter, 2007). However, its performance can vary significantly depending on the plant species, tissue type, developmental stage, and environmental conditions (Sunilkumar et al., 2002). For example, the CaMV35S promoter exhibits poor activity in many monocots and can be silenced in certain stable transgenic lines due to epigenetic regulation (Al-Kaff et al., 1998; Mette et al., 2000; Potenza et al., 2004; Okumura et al., 2016). Additionally, overexpression of foreign genes under the CaMV35S promoter may cause unintended phenotypic changes or metabolic burden, especially in whole plants (Schubert et al., 2004; Peremarti et al., 2010).

Promoters derived from plant ubiquitin or actin genes, such as ZmUbi-1and OsAct-1, tend to offer more consistent and robust expression across a wider range of species, including both dicots and monocots (McElroy et al., 1990; Christensen and Quail, 1996; Hernandez-Garcia and Finer, 2014). These promoters are frequently used in cereal crops and model systems like rice and *Brachypodium* (Sivamani and Qu, 2006; Hong et al., 2008). However, even these promoters are not universally optimal: their activity can still be influenced by positional effects upon integration, chromatin context, or methylation-induced silencing (Matzke and Matzke, 1998; Mette et al., 2000). Another limitation of natural promoters is their lack of modular control. Most natural constitutive promoters drive unregulated expression, which may not be desirable for certain applications, especially when the recombinant protein is toxic to the host cells, energetically costly to produce, or requires precise temporal or spatial expression (Venter, 2007; Liu and Stewart, 2016). Similarly, the use of natural tissue-specific promoters, while offering localization of expression (e.g., seed-specific or leaf-specific promoters), is often constrained by their limited strength and narrow activity range (Peremarti et al., 2010; Hernandez-Garcia and Finer, 2014). Furthermore, native promoters often contain cis-regulatory elements whose functions are not fully understood. This complexity can complicate efforts to fine-tune expression levels or engineer predictable promoter performance across different systems.

While natural promoters provide a useful starting point for recombinant gene expression in plants, their limitations, including species- and tissue-specific variability, susceptibility to silence, and lack of tunability, underscore the need for more rational and robust alternatives.

### 3.2 Synthetic promoters: rational design and *de novo* construction

Synthetic promoters are constructed by assembling defined regulatory elements, such as enhancers, core promoter motifs, and transcription factor binding sites, in a modular fashion to achieve precise control over gene expression (Khan and Lister, 2025; Nayak et al., 2025). Synthetic promoters offer several advantages over their natural counterparts, including enhanced strength, reduced susceptibility to epigenetic silencing, minimized sequence redundancy, and the ability to program specific spatial or temporal expression profiles (Cai et al., 2020; Yasmeen et al., 2023) (**Table 2**).

**Table 2:** Synthetic and engineered promoters for enhanced recombinant protein expression in plants.

Promoter	Design features	Relative strength	Regulation type	Advantages	Limitations	References
Double CaMV35S (2×35S)	Tandem duplication of CaMV35S enhancer region	Very high (in dicots)	Constitutive	Stronger than native CaMV35S; widely used in dicots	Can still be silenced; less effective in monocots	(Kay et al., 1987; Benfey and Chua, 1990; Venter, 2007; Liu and Stewart, 2016)
Super promoter (e.g., 4×35S)	Fusion of multiple enhancer elements from CaMV35S and other viruses	Very high	Constitutive	Enhanced activity across a wide range of tissues	High expression may trigger silencing or metabolic burden	(Jiang et al., 2019; Gorripati et al., 2021)
G-box-enhanced promoters	Incorporation of G-box motifs (e.g., from Arabidopsis genes)	Moderate to high	Constitutive / Inducible	Responsive to light or hormones; modular tuning	May require specific transcription factors	(Bovy et al., 1995; Liu and Stewart, 2016)
XVE system (Estrogen-inducible)	Fusion of LexA operator, VP16 activator, and estrogen receptor	Off until induced	Chemically inducible	Tight regulation; low background expression	Requires estrogen application; more complex system	(Zuo et al., 2000; Brand et al., 2006; Corrado and Karali, 2009)
GVG system (Dexamethasone-inducible)	Chimeric promoter with GAL4, VP16, and GR domains	Off until induced	Chemically inducible	Strong induction; temporal control	Requires dexamethasone; possible off-target effects	(Aoyama and Chua, 1997; Brand et al., 2006)
Heat shock promoter derivatives	Engineered with optimized heat shock elements	Low to high (heat-dependent)	Physically inducible (heat)	Environmentally responsive	Limited control over induction level; stress effect on host	(Schoffl et al., 1984; Hernandez-Garcia and Finer, 2014; Liu and Stewart, 2016)
BiGSSP series (BiGSSP2, 3, 6, 7)	Bidirectional synthetic promoters combining green tissue-specific cis-elements	Comparable to or higher than CaMV35S in green tissues	Tissue-specific (green)	Enables simultaneous high expression of two genes; reduces need for multiple promoters	Limited activity in non-green tissues	(Bai et al., 2020)
A27znGlb1 promoter	Chimeric maize *27zn* and *Glb1* promoter domains	Strong in maize kernel endosperm and embryos	Tissue-specific (seed)	Efficient seed-specific production; avoids off-target expression	Limited to seed tissue; monocot-biased	(Shepherd and Scott, 2009)
E8-E4 hybrid promoter	Combination of tomato *E8* and *E4* ripening gene promoters	Strong during fruit ripening	Developmental stage-specific (fruit ripening)	Targeted expression during ripening; useful for metabolic engineering in fruit	Not active before ripening; species-specific	(Bestwick and Kellogg, 2000)
Tri-hybrid MFH17	Engineered fusion of MMV, FMV, HRLV pararetrovirus promoter domains	Exceeds enhanced 2×35S	Constitutive (broad-spectrum)	Very high activity in *N. benthamiana* and other species; good for transient expression	Stability and regulation in long-term cultures not fully tested	(Sherpa and Dey, 2024)
Bidirectional ZmUbi1-based promoters	Derived from maize Ubi-1 promoter arranged bidirectionally	High in green tissues; strong in cereals	Tissue-specific (green, monocot)	Coordinates multi-gene expression in crops	Limited performance in dicots	(Kumar et al., 2015)

A typical synthetic promoter consists of a minimal core promoter, often containing a TATA box and TSS, combined with multiple upstream activating sequences (UAS) or enhancer elements (Cai et al., 2020). For example, the use of tandem repeats of enhancer motifs from the CaMV35S promoter or the octopine synthase (Ocs) promoter has yielded synthetic constructs with significantly stronger transcriptional activity than the original promoters (Fromm et al., 1989; Ni et al., 1995; Ali and Kim, 2019). A well-known synthetic construct is the “Super promoter,” which combines four tandem repeats of the CaMV35S enhancer with a minimal promoter, resulting in up to 10-fold higher expression in transient assays compared to the native CaMV35S promoter (Kay et al., 1987). Moreover, synthetic promoters can be tailored for inducibility or orthogonality, allowing them to function independently of endogenous regulatory networks (Nemhauser and Torii, 2016). This is especially valuable for applications in synthetic biology, where multiple transgenes must be expressed independently or in a coordinated manner.

One special class of synthetic promoters is hybrid promoters, which are created by fusing regulatory elements from different natural promoters to combine desirable features, such as strong constitutive activity, tissue specificity, or inducibility, into a single expression cassette (Venter, 2007; Liu and Stewart, 2016). For instance, enhancer sequences from viral or highly active plant promoters (e.g., CaMV35S or ZmUbi-1) can be combined with tissue-specific minimal promoters to enhance expression in a defined context (Potenza et al., 2004; Ali and Kim, 2019). A practical example includes the fusion of the CaMV35S enhancer to the rice glutelin promoter to generate a hybrid promoter that drives high-level, seed-specific expression, useful for expressing storage proteins or pharmaceutical compounds in edible plant tissues (Wu et al., 2000; Qu le and Takaiwa, 2004). Similarly, hybrid constructs that incorporate stress-responsive elements into constitutive promoters can enable upregulated expression under specific conditions, such as pathogen attack or nutrient deficiency (Hernandez-Garcia and Finer, 2014; Ali and Kim, 2019).

The design of synthetic promoters increasingly leverages high-throughput screening and computational tools (Brophy and Voigt, 2014; Zeng et al., 2025). Libraries of synthetic promoter variants with different combinations or numbers of cis-elements can be screened using reporter genes (e.g., GFP, GUS, luciferase) in transient expression systems like *Nicotiana benthamiana* or protoplast assays (Ali and Kim, 2019; Yasmeen et al., 2023). Quantitative data from these screens are used to identify promoter sequences that confer optimal expression levels. Machine learning models trained on experimental datasets have also been used to predict promoter activity and guide the design of novel promoter sequences with desired features (Vaishnav et al., 2022; Wang et al., 2025). This data-driven promoter engineering approach offers the potential for faster and more reliable generation of promoters tailored to specific plant species, tissues, or production needs.

### 3.3 Comparative performance and practical trade-offs

While both natural and synthetic promoters have been widely applied in plant molecular farming, their relative strengths and limitations become apparent when evaluated across different species and production platforms (Venter, 2007; Liu and Stewart, 2016). Natural promoters, particularly those derived from endogenous housekeeping genes, often exhibit superior long-term stability and regulatory acceptance, especially in stable transgenic plants and plant cell cultures (Hernandez-Garcia and Finer, 2014; Ali and Kim, 2019). However, their transcriptional strength and tunability are typically constrained by evolutionary regulatory logic and native chromatin context (Liu and Stewart, 2016). In contrast, synthetic promoters enable programmable expression levels, orthogonality, and inducibility, making them particularly attractive for transient expression systems and multigene constructs (Belcher et al., 2020; Cai et al., 2020). These advantages come at the cost of increased context dependency, as synthetic promoters frequently display variable activity across species, tissues, or chromatin environments (Yasmeen et al., 2023; Nayak et al., 2025). Moreover, excessive stacking of enhancer motifs can trigger epigenetic silencing or diminishing returns in transcriptional output (Rajeevkumar et al., 2015; Buitrago et al., 2026). Consequently, promoter selection in practical applications increasingly reflects a compromise between strength, predictability, regulatory robustness, and scalability rather than maximal expression alone.

## 4 Promoter functionality: constitutive, inducible, and tissue-specific regulation

In terms of function and regulation, promoters are classified as constitutive, inducible, or tissue-specific (spatiotemporal) (Hernandez-Garcia and Finer, 2014; Ali and Kim, 2019). While constitutive promoters have driven major progress in plant-based recombinant protein production, their continuous activity can cause cytotoxicity, metabolic stress, or developmental disruption (Streatfield, 2007; Liu and Stewart, 2016). In contrast, inducible and tissue-specific promoters allow precise spatial and temporal control of gene expression, making them better suited for proteins such as hormones, signaling peptides, or enzymes that require tightly regulated expression (**Table 3**).

**Table 3:** Summary of inducible and tissue-specific promoter types in plant systems.

Promoter type	Subtype/Example	Key features	Applications	References
Inducible	Chemical-inducible (e.g., Tetracycline, Dexamethasone, Ethanol)	Triggered by external compounds; reversible	On-demand protein expression during specific growth phases	(Gatz, 1997; Tang et al., 2004; Bortesi et al., 2012; Dugdale et al., 2013)
	Physical-inducible (e.g., heat-shock, light)	Activated by temperature or light; spatiotemporal control	Transient expression under defined environmental cues	(Chinnusamy et al., 2007; Omelina et al., 2022; Xu et al., 2024)
	Developmental stage-specific (e.g., senescence promoters)	Expression tied to growth stage	Delayed expression to reduce early growth effects	(Noh and Amasino, 1999; Quirino et al., 2000)
Tissue- or organelle-specific	Seed-specific (e.g., glutelin, glycinin)	Stable, high-protein storage; low water content	Vaccines, nutraceuticals, long-term storage	(Takaiwa et al., 2007; Aoyagi et al., 2018)
	Leaf-specific (e.g., Rbsc)	High expression in chloroplast-rich tissues	Transient expression in *N. benthamiana*	(Alotaibi, 2021; Boruah et al., 2023)
	Root/tuber-specific (e.g., patatin)	Expression in underground organs	Edible vaccines, biofactories	(Liu et al., 1990; Noh et al., 2012)
	Chloroplast promoters (e.g., psbA)	High protein yield; polycistronic; maternal inheritance	High-level production with reduced transgene escape	(Maliga, 2004; Rasala et al., 2011)

### 4.1 Constitutive promoters

Constitutive promoters drive continuous gene expression in virtually all tissues and developmental stages, independent of environmental or physiological cues (Potenza et al., 2004; Venter, 2007). They have been instrumental in advancing plant-based recombinant protein production by ensuring robust and reliable transgene expression (Schillberg et al., 2013). Common examples include the CaMV35S promoter and its enhanced variants, as well as plant-derived promoters such as ubiquitin or actin promoters (Cornejo et al., 1993; Norris et al., 1993; Christensen and Quail, 1996). Despite their simplicity and high activity, constitutive promoters can impose metabolic and physiological burdens on host cells, especially when expressing cytotoxic or resource-intensive proteins (Streatfield, 2007). Continuous overexpression may disrupt normal cellular homeostasis, leading to growth retardation or reduced viability (Liu and Stewart, 2016; Ali and Kim, 2019). Consequently, while constitutive promoters remain the workhorse for proof-of-concept studies and high-yield production of benign proteins, their use requires careful optimization or combination with regulatory elements to balance productivity and host health.

### 4.2 Inducible promoters

Inducible promoters allow precise control of gene expression in response to specific stimuli. Chemical-inducible systems (e.g., tetracycline, dexamethasone, ethanol) are widely used due to their tight regulation and reversibility (Corrado and Karali, 2009; Gatz, 2013). These systems enable temporal activation of transgenes during optimal production windows, minimizing stress on the host. Physical inducers such as heat or light offer environmental control but may be limited by application constraints in dense cultures (Ochoa-Fernandez et al., 2020; Xu et al., 2024). Developmental promoters restrict expression to specific stages (e.g., leaf senescence), reducing metabolic burden during early growth (Fischer et al., 2012; Sherpa and Dey, 2024) (**Table 3**).

### 4.3 Tissue-specific promoters

Tissue-specific promoters confine transgene expression to defined organs or cell types, enabling targeted protein accumulation and reducing systemic effects (Potenza et al., 2004; Villao-Uzho et al., 2023). Seed-specific promoters provide a stable production environment and ease of storage (Stoger et al., 2005; Boothe et al., 2010). Leaf- and root-specific promoters support localized expression in vegetative or underground tissues, respectively (Benfey and Chua, 1990; Yamamoto et al., 1991). Trichome-specific promoters compartmentalize metabolite synthesis (Lange et al., 2000). Additionally, chloroplast-targeted expression using plastid-specific promoters enables exceptionally high protein yields and added biosafety via maternal inheritance (Maliga, 2004; Sinagawa-Garcia et al., 2009) (**Table 3**).

### 4.4 Challenges with inducible and tissue- and organelle-specific promoters

While inducible and tissue-specific promoters offer critical benefits, their practical application faces certain challenges. Inducer toxicity, leaky expression, variable penetration in tissues, or high cost of chemical inducers can limit field or industrial applications (Zuo and Chua, 2000; Corrado and Karali, 2009; Gatz, 2013). Moreover, the strength of tissue-specific promoters may not always be sufficient for commercial-scale protein accumulation (Stoger et al., 2005; Wrighton, 2018). Ongoing research focuses on engineering synthetic inducible promoters with tighter control, improved dynamic range, and reduced basal activity (Schaumberg et al., 2016). New biosensors and synthetic transcriptional circuits, inspired by microbial and mammalian synthetic biology, are being adapted for plant systems to build complex regulation schemes, including feedback loops, Boolean logic gates, and multiplex control over multiple genes (Brophy and Voigt, 2014; Schaumberg et al., 2016; Huang et al., 2021).

## 5 Applications of promoter engineering across plant-based production platforms

**Figure 3:**
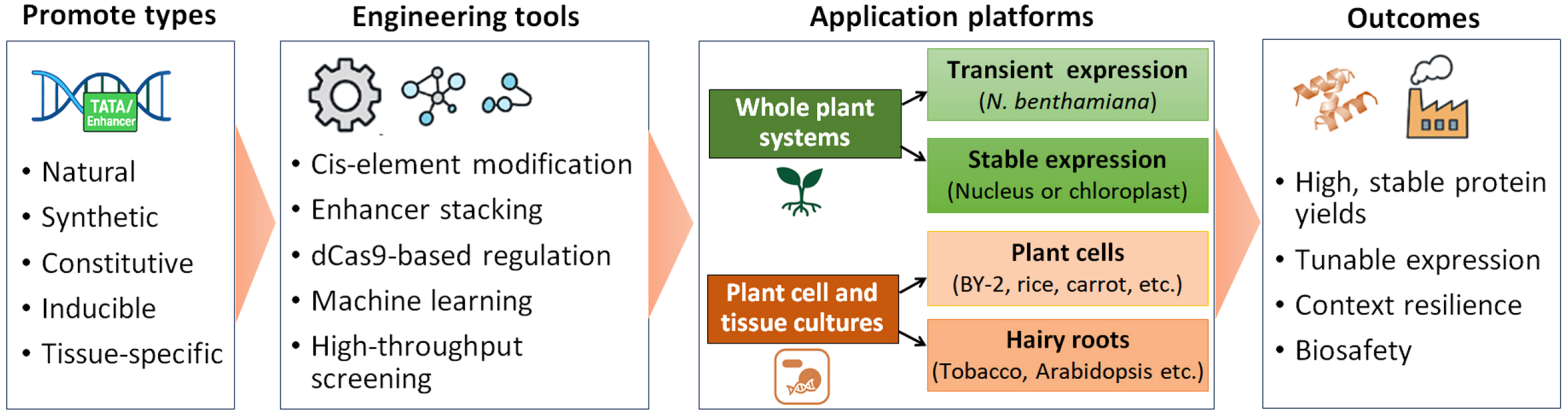
Promoter engineering strategies for plant molecular farming. Diverse promoter types are optimized using synthetic biology tools and computational design, enabling enhanced and predictable recombinant protein expression across multiple plant-based production platforms.

Plant molecular farming relies heavily on precise and robust control of transgene expression (Eidenberger et al., 2023). Promoter engineering plays a central role in optimizing gene expression across diverse plant-based production systems (**Figure 3**). Each platform, whether whole plant stable expression, transient expression, or plant cell and tissue cultures, presents unique opportunities and challenges (Schillberg and Finnern, 2021). By tailoring promoter activity to the specific characteristics of each platform, researchers can significantly enhance recombinant protein yields, stability, and consistency, while mitigating issues such as gene silencing, developmental interference, or metabolic stress (Feng et al., 2022) (**Table 4**). A comparative overview of promoter classes, highlighting their relative strengths, limitations, and suitability across plant molecular farming platforms, is provided in **Table 5**.

**Table 4:** Promoter engineering strategies used in different plant-based production platforms.

Platform	Challenges addressed	Promoter engineering strategies	Representative examples	Literature
Transient expression systems	Rapid production, multi-gene coordination, expression tuning	- Strong synthetic viral promoters - Orthogonal promoters (TALEs, CRISPRa) - Ratio tuning for multi-subunit genes	- 2× or 4× CaMV35S - CPMV-based promoters - Synthetic promoters for light/heavy chain balance in mAbs	(Gleba et al., 2005; Venter, 2007; Peyret et al., 2019; Efremova et al., 2020)
Stable transgenic plants - nuclear expression	Gene silencing, positional effects, developmental regulation	- Hybrid promoters (viral enhancers + plant core) - Tissue-specific promoters - Insulators and S/MARs	- CaMV35S-FMV “super promoter” - FMV-Ubi fusion - Rice glutelin promoter for seeds	(Leisy et al., 1990; Christensen and Quail, 1996; Kumar et al., 2011; Kummari et al., 2020)
Stable transgenic plants -plastid expression	Polycistronic expression, plastid-specific regulation, containment	- Engineered plastid promoters with 5′- and 3′- UTRs - High-yield transcriptional units	- psbA, rrn promoters - Transplastomic tobacco and lettuce expressing up to 70% TSP	(Klein et al., 1992; Eibl et al., 1999; Daniell et al., 2009; Drechsel and Bock, 2011; Fatima et al., 2025)
Plant cell and tissue cultures	Low expression, gene silencing, secretion efficiency	- Housekeeping gene promoters (Ubi, Act, EF1α, α-tubulin) - IME elements - Synthetic promoters	- BY-2 and rice-specific synthetic promoters - Moss promoters for α-galactosidase and factor H	(Buttner-Mainik et al., 2011; Reski et al., 2015; Jores et al., 2021; Nguyen et al., 2024)

CPMV, cowpea mosaic virus promoter; FMV, figwort mosaic virus promoter; IME, intron-mediated enhancement.

**Table 5:** Comparison of promoter types across plant molecular farming platforms.

Promoter type	Typical strength	Predictability / stability	Scalability & industrial suitability	Platform suitability	Key advantages	Key limitations / trade-offs
Viral constitutive promoters (e.g., CaMV35S, FMV)	High (short term)	Low–moderate (prone to silencing in stable systems)	Moderate (regulatory concerns; instability in long-term use)	Transient expression; short-term stable expression	Strong activity; well characterized; rapid expression	Species bias; epigenetic silencing; regulatory scrutiny; poor long-term stability
Endogenous constitutive promoters (e.g., ubiquitin, actin, EF1α)	Moderate–high	High (especially in stable lines and cell cultures)	High (regulatory-friendly; reproducible performance)	Stable transgenic plants; plant cell cultures	Long-term stability; reduced silencing; cross-species utility	Limited tunability; less maximal expression than viral promoters
Synthetic promoters	Tunable (low–very high)	Variable; context dependent	Moderate (screening and validation required)	Transient systems; synthetic circuits; multigene expression	Programmable strength; orthogonality; modular design	Platform-specific behavior; diminishing returns from motif stacking
Hybrid promoters (natural + synthetic elements)	High	Moderate–high	High (balanced performance)	Stable plants; cell cultures; tissue-specific expression	Combines strength and stability; flexible design	Design complexity; still context sensitive
Inducible promoters (chemical/physical)	Moderate–high (on induction)	High when optimized	Moderate (inducer cost, delivery constraints)	Cell cultures; controlled whole-plant systems	Temporal control; reduced metabolic burden	Leaky expression; inducer toxicity; scale-up challenges
Tissue-specific promoters	Low–moderate	High within target tissue	High for specialty products	Seeds, leaves, roots, trichomes	Spatial control; reduced pleiotropy	Lower expression levels; limited generalizability
Plastid promoters (e.g., psbA, rrn)	Very high	Very high	High (contained expression)	Chloroplast transformation	Extreme protein yields; transgene containment	Limited species applicability; specialized transformation

### 5.1 Transient expression systems

Transient expression systems, especially in *N. benthamiana*, have emerged as a rapid and high-yielding method for recombinant protein production. These systems are ideal for prototyping synthetic promoter constructs and for large-scale protein production within a short time frame (often within 7–10 days) (Leuzinger et al., 2013; Peyret and Lomonossoff, 2015). Promoter engineering in transient systems emphasizes not only strength, but also predictable response dynamics, multi-gene expression balance, and orthogonality (Gelvin, 2003; Rybicki, 2020). Strong synthetic promoters based on viral enhancers are commonly used to maximize expression in agroinfiltrated leaves (Sanger et al., 1990; Shepherd and Scott, 2009; Efremova et al., 2020; Kumari et al., 2024; Sherpa and Dey, 2024). Orthogonal synthetic promoters, such as those controlled by artificial transcription factors (e.g., TALEs, CRISPRa), are gaining traction for coordinated expression of multi-subunit proteins, such as virus-like particles or antibody assemblies (Kar et al., 2022; Yasmeen et al., 2023; Majdi et al., 2025). In addition, promoter engineering enables fine-tuning of expression ratios between co-expressed genes (e.g., light and heavy chains of monoclonal antibodies) to ensure correct folding and assembly, which is critical for product quality (Sainsbury and Lomonossoff, 2008; Niemer et al., 2014; Brooks et al., 2023).

### 5.2 Stable transgenic plants - nuclear transformation

Stable transgenic plants offer a cost-effective and scalable platform for long-term production of recombinant proteins (Xu et al., 2016; Schillberg and Finnern, 2021). However, transgene expressions in these systems are often influenced by positional effects, epigenetic silencing, and developmental cues (Rajeevkumar et al., 2015; Cao and Chen, 2024; Talarico et al., 2024). Promoter engineering has proven to be an effective approach to address these limitations. Engineered promoters that combine strong viral enhancers (e.g., CaMV35S, FMV, or MMV) with minimal plant-derived promoters have shown enhanced transcriptional strength and reduced susceptibility to silencing (Sanger et al., 1990; Yasmeen et al., 2023; Nayak et al., 2025). For instance, hybrid promoters such as the “super promoter” or FMV-Ubi (ubiquitin) fusions have driven robust, constitutive expression across multiple plant species (Ni et al., 1995; Tavva et al., 2006; Lee et al., 2007; Ali and Kim, 2019).

In crops such as rice, maize, or soybean, tissue-specific engineered promoters are commonly used to express proteins in edible organs (e.g., seeds or tubers), reducing processing costs and enabling oral delivery for vaccines and nutraceuticals (Takeyama et al., 2015; Schillberg and Finnern, 2021; Shi et al., 2025). Additionally, synthetic insulator sequences and scaffold/matrix attachment regions (S/MARs) are increasingly incorporated alongside engineered promoters to buffer against chromatin position effects and enhance transgene stability across generations (Dolgova and Dolgov, 2019; Kurbidaeva and Purugganan, 2021).

### 5.3 Stable transgenic plants - plastid transformation

Promoter engineering is also critical in plastid (chloroplast) expression systems, where expression is often polycistronic and driven by distinct transcriptional machinery (Daniell et al., 2016; Yang et al., 2022). Strong plastid promoters such as psbA and rrn have been engineered with 5′- and 3′-UTRs to enhance translation efficiency and transcript stability (Klein et al., 1992; Eibl et al., 1999; Drechsel and Bock, 2011). Promoter engineering in plastids enables very high levels of recombinant protein accumulation, sometimes reaching up to 70% of total soluble protein (TSP) while avoiding transgene escape via pollen (Ruf et al., 2001; Daniell et al., 2009). This has been exploited to produce vaccines, enzymes, and antimicrobial peptides in transplastomic tobacco and lettuce (Verma and Daniell, 2007; Ruhlman et al., 2010; Lakshmi et al., 2013; Fatima et al., 2025).

### 5.4 Plant cell and tissue cultures

This platform comprises two *in vitro* culture systems: plant cell suspensions and hairy roots. Among them, plant cell suspension culture, including moss, is the dominant system (Xu et al., 2025), whereas only a few reports have described recombinant protein production in hairy roots (Zhang et al., 2019). Plant cell suspension cultures, such as tobacco, rice, carrot cells, and moss (*Physcomitrium patens)*, provide a clean, contained, and scalable environment for producing biopharmaceuticals under GMP-compliant conditions (Xu et al., 2011; Xu and Zhang, 2014). Among these, tobacco Bright Yellow-2 (BY-2) cells have gained increasing attention due to their rapid proliferation rate, scalability in bioreactors, and suitability for downstream processing (Xu et al., 2025). Notably, BY-2 cells are the only plant-based platform to have successfully produced FDA-approved therapeutic proteins, Elelyso^®^ and Elfabrio^®^ by Protalix (Israel) (Schiffmann et al., 2019; Germain and Linhart, 2024). This achievement demonstrates the feasibility of plant cell-based systems for clinical-grade protein production and underscores the untapped potential of BY-2 cells as a robust and scalable biomanufacturing platform. However, their broader adoption of commercial biopharmaceutical production remains limited. A major technical bottleneck is the low and inconsistent levels of recombinant protein expression, largely due to the continued reliance on heterologous promoters such as CaMV35S, which frequently results in unstable or silenced expression during long-term culture (Schillberg et al., 2019; Xu et al., 2025).

In plant cell suspension cultures, promoter behavior differs substantially from that observed in differentiated tissues, underscoring the need for platform-specific promoter engineering. Undifferentiated cells undergo rapid division and prolonged passaging or subcultures, conditions that exacerbate epigenetic drift, transcriptional variability, and promoter methylation (Matzke and Mosher, 2014; Xu et al., 2025). Viral promoters such as CaMV35S, while effective in transient or short-term systems, often exhibit progressive transcriptional silencing during long-term culture, resulting in declining recombinant protein yields (Gelvin, 2003; Kirchhoff et al., 2012; Schillberg et al., 2019). In contrast, promoters derived from constitutively expressed endogenous housekeeping genes, such as ubiquitin, actin, EF1α, and rice α-tubulin, have demonstrated comparatively stable transcriptional activity over months of continuous passaging in systems such as tobacco BY-2 and rice suspension cells (Christensen and Quail, 1996; Park et al., 2010; Li, 2014; Shaul, 2017). These promoters appear better adapted to the transcriptional landscape of proliferative cells and are less prone to silencing, making them more suitable for industrial-scale, GMP-compliant production processes.

Despite these advances, significant challenges remain in optimizing promoter performance in plant cell cultures. Transcriptional strength alone does not guarantee stable protein accumulation, as excessive expression can activate post-transcriptional gene silencing or impose metabolic and secretory stress on rapidly dividing cells (Fischer et al., 2015; Schillberg et al., 2019). Furthermore, promoter activity may fluctuate with culture age, nutrient availability, and bioreactor conditions, complicating process consistency and scalability (Xu et al., 2011; Buyel et al., 2017). These factors highlight the importance of balancing promoter strength with long-term expression stability and cellular fitness rather than maximizing transcription alone (Tuse et al., 2014).

## 6 Species- and platform-dependent promoter behavior in plant molecular farming

**Figure 4:**
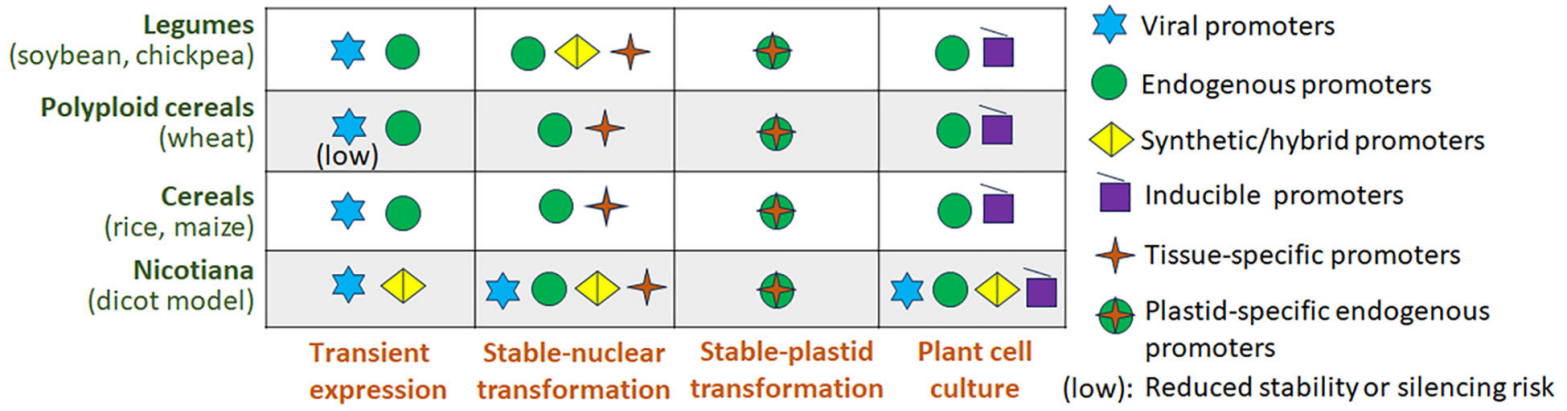
Schematic comparison of promoter class suitability across crop species and plant-based production platforms. The figure summarizes relative promoter performance in terms of expression strength, stability, and context dependence in transient expression systems, stable transgenic plants, plant cell cultures, and plastid-based platforms.

Although promoter engineering strategies are frequently developed and benchmarked in model systems, their performance in plant molecular farming is highly dependent on species-specific genomic contexts and production platforms, with significant implications for the translational efficiency and robustness of these strategies across different crop species. **Figure 4** provides a schematic overview of promoter class suitability across major crop groups and production platforms, highlighting key trade-offs relevant to translational applications.

### 6.1 Cross-species promoter performance

Promoter performance varies markedly across plant species and correlates strongly with both genome composition and transformation platform. In model dicots such as *N. benthamiana*, viral promoters including CaMV35S and its enhanced derivatives routinely drive very high transient expression, often yielding recombinant proteins in the range of 0.1–5% of TSP within days of agroinfiltration (Chen et al., 2011; Buyel et al., 2017; Akher et al., 2025). In contrast, the same promoters frequently exhibit reduced activity or progressive transcriptional silencing in stable transformants of cereals, particularly in polyploid crops such as wheat, where epigenetic repression and homology-dependent silencing are more pronounced (Mette et al., 2000; Meyer, 2015). In monocot systems, plant-derived promoters such as ZmUbi-1 or OsAct-1 consistently outperform viral promoters, supporting both higher transformation efficiency and more stable long-term expression (McElroy et al., 1990; Christensen and Quail, 1996). These trends underscore that promoter strength observed in Nicotiana transient assays cannot be directly extrapolated to stable expression in cereal or legume crops.

Legume crops, such as soybeans and cowpea often exhibit intermediate behavior between *Nicotiana* and cereals with respect to promoter performance. While viral promoters can drive strong expression in transient or early stable expression phases, long-term stability is frequently improved when endogenous or hybrid promoters are used (Hernandez-Garcia et al., 2010; Hernandez-Garcia and Finer, 2014). For example, ubiquitin- and actin-based promoters have been reported to provide more consistent expression in soybean and chickpea compared with CaMV35S, particularly in seed- or tissue-specific applications (Hernandez-Garcia et al., 2009; Das Bhowmik et al., 2019; Singh et al., 2022). These observations highlight that genome complexity, epigenetic regulation, and transformation protocol collectively shape promoter performance, reinforcing the need for crop-specific promoter optimization rather than reliance on a universal “strong” promoter (Mette et al., 2000; Meyer, 2015).

### 6.2 Production platform-dependent promoter performance

Promoter performance in molecular farming is strongly platform dependent, and activity observed in one system often fails to translate directly to another (Butaye et al., 2004; Hernandez-Garcia and Finer, 2014). Transient expression platforms, particularly agroinfiltration in Nicotiana, favor viral and synthetic promoters that drive rapid, high-level transcription under minimal chromatin constraint, making them effective for screening and short-term production but poor predictors of long-term stability (Tuse et al., 2014; Buyel et al., 2017). In contrast, stable nuclear transformation exposes promoters to chromatin integration and epigenetic regulation, where viral promoters frequently show variable expression or silencing, especially in cereals and polyploid crops, while endogenous or hybrid promoters provide more predictable long-term expression at moderate strength (Meyer, 2015; Altpeter et al., 2016). Plant cell cultures impose additional constraints due to rapid cell division and prolonged passaging, favoring endogenous promoters such as ubiquitin or EF1α that sustain transcription over time (Hernandez-Garcia and Finer, 2014). Plastid expression systems enable exceptionally high and stable expression insulated from nuclear epigenetic effects but remain limited in species range and regulatory flexibility (Maliga, 2004; Bock, 2014). Together, these differences highlight the need to align promoter design with platform-specific constraints rather than relying on promoter strength alone.

### 6.3 Transformation efficiency and genotype dependence

Reported transformation efficiencies further illustrate the context dependence of promoter choice. In Nicotiana species, *Agrobacterium*-mediated transformation efficiencies commonly exceed 20–40%, enabling rapid screening of synthetic and viral promoters (Gelvin, 2003). By contrast, transformation efficiencies in major cereal crops are substantially lower and genotype dependent, often ranging from 1–10% in rice and maize and frequently below 1% in wheat (Jones et al., 2005; Ishida et al., 2007; Altpeter et al., 2016). Under these constraints, promoters that minimize transgene silencing and developmental penalties become critical, as the cost of screening multiple independent events is substantially higher (Mette et al., 2000; Meyer, 2015). Endogenous constitutive promoters such as ubiquitin, EF1α, or tubulin not only support more reliable expression but also reduce the risk of promoter-induced growth defects that can negatively affect regeneration and recovery of transformed plants, particularly in recalcitrant genotypes (Holtorf et al., 1995; Christensen and Quail, 1996).

### 6.4 Quantitative effects of promoter selection across plant expression platforms

Several quantitative case studies further highlight how promoter choice directly influences recombinant protein yield across diverse plant platforms. In *N. benthamiana*, the CaMV35S promoter routinely drives transient expression levels exceeding 1–3 g/kg fresh weight for antibodies and vaccine antigens when combined with deconstructed viral vectors such as the magnICON system, whereas native constitutive promoters such as actin or ubiquitin typically achieve only 10–25% of this level in direct comparative assays (Gleba et al., 2005; Giritch et al., 2006; Gleba et al., 2007; Lomonossoff and D’Aoust, 2016; Buyel et al., 2017). In stably transformed cereals, overall promoter performance is markedly lower. The ZmUbi-1 generally drives recombinant protein accumulation at approximately 0.1–0.5% of TSP in maize and barley, while the OsAct-1 yields only 0.01–0.05% TSP in rice callus or suspension cultures, reflecting strong positional effects and transcriptional silencing associated with stable nuclear integration (Christensen et al., 1992; Cornejo et al., 1993; Stoger et al., 2005; Takaiwa et al., 2007). In legumes such as soybean, seed-specific promoters including β-conglycinin and glycinin enable higher and more stable expression, producing recombinant proteins at levels ranging from 0.1 to 2% of seed dry weight. In contrast, constitutive promoters such as CaMV35S rarely exceed 0.01–0.03% in vegetative tissues due to developmental regulation and promoter silencing effects (Hood et al., 1997; Moravec et al., 2007; Schmidt and Herman, 2008).

### 6.5 Implications for translational promoter selection

Cross-species comparisons and platform-specific studies demonstrate that promoter strength and stability vary substantially across crop species, tissue types, and transformation contexts and therefore cannot be considered in isolation. Promoter selection must be evaluated alongside transformation efficiency, ploidy level, epigenetic landscape, and the intended production platform. Promoters optimized in Nicotiana transient expression systems are highly effective for rapid prototyping and short-term protein production but should not be assumed to perform equivalently in stable transgenic plants, cereals, legumes, or plant cell cultures without rigorous revalidation (Tuse et al., 2014; Buyel et al., 2017). For translational and industrial applications, particularly in polyploid crops, promoter stability, epigenetic compatibility, and regeneration fitness are often more predictive of long-term success than maximal transcriptional output (Meyer, 2015; Altpeter et al., 2016). Accordingly, endogenous and hybrid promoters generally provide more reliable and durable expression across species and platforms, whereas viral and highly synthetic promoters exhibit increased susceptibility to silencing and context dependence (Butaye et al., 2004; Hernandez-Garcia and Finer, 2014).

In this translational context, synthetic and machine learning–designed promoters offer attractive tunability and modularity but remain constrained by training-data bias and limited validation beyond model systems (Camacho et al., 2018; Zrimec et al., 2020). Most reported successes rely on transient expression assays, protoplasts, or massively parallel reporter assays in a small number of species, conditions that do not fully capture the chromatin, developmental, and epigenetic constraints encountered in stable transgenic crops or long-term production platforms (Washburn et al., 2019; Jores et al., 2021). As a result, while synthetic and machine learning-based approaches represent powerful tools for promoter discovery and rapid prototyping, their real-world performance and scalability in plant molecular farming remain less predictable than those of endogenous or hybrid promoters. Together, these considerations underscore the need to move beyond one-size-fits-all promoter usage toward rational, species- and platform-specific promoter engineering strategies that prioritize robustness, reproducibility, and translational reliability over peak expression levels.

## 7 Promoter silencing mechanisms and strategies to mitigate silencing

One of the major challenges in achieving stable transgene expression in plant molecular farming is gene silencing, which can dramatically reduce or abolish recombinant protein accumulation over time (**Table 6**) (Garcia-Perez et al., 2025; Xu et al., 2025). Two primary mechanisms are recognized: transcriptional gene silencing (TGS) and post-transcriptional gene silencing (PTGS). TGS typically involves DNA methylation of promoter regions and the formation of heterochromatin, which blocks transcription initiation (He et al., 2022; Leichter et al., 2022). This is frequently observed in transgenes driven by viral promoters, such as CaMV35S, especially when present in multiple copies or arranged in tandem repeats (Rajeevkumar et al., 2015; Okumura et al., 2016). PTGS, on the other hand, operates through RNA interference (RNAi) pathways and is triggered by the formation of aberrant or double-stranded RNAs, often resulting from high levels of transgene expression or read-through transcription (Chicas and Macino, 2001; Hoffer et al., 2011). These RNAs are processed by Dicer-like enzymes into small interfering RNAs (siRNAs), which guide the RNA-induced silencing complex (RISC) to degrade complementary mRNA transcripts, thus suppressing protein accumulation (Carthew and Sontheimer, 2009). Both TGS and PTGS can be heritable and are influenced by the transgene’s genomic context, promoter origin, and expression load (Guo et al., 2016).

**Table 6:** Promoter silencing and expression stability in transgenic plant lines.

Promoter	Type	Silencing susceptibility	Expression stability	Factors influencing stability	Strategies to improve stability
CaMV35S	Native viral	High (especially in long-term or high-copy lines)	Variable; often declines over generations	Homology-dependent gene silencing (HDGS); DNA methylation; copy number	Use minimal/modified versions; single-copy insertion; avoid repeats
2×35S/4×35S promoters	Synthetic (viral enhancer-based)	Very high	Often unstable in stable transgenics	Strong expressions may trigger PTGS; tandem repeats enhance risk	Combine with S/MARs; inducible expression
Ubiquitin	Native plant	Moderate	Generally stable	Host species; promoter isoform	Use endogenous (species-matched) versions; test multiple constructs
EF1α	Native plant	Low to moderate	Relatively stable in many systems	Developmental regulation; positional effects	Combine with insulator sequences or S/MARs
RbcS	Native plant (tissue-specific)	Low	High (in green tissues)	Strong but tissue-limited; less prone to silencing	Best for leaf or green tissue-specific expression
Actin	Native plant	Moderate	Fairly stable	Activity may vary during development	Use endogenous version; test multiple insertion events
XVE/GVG (Inducible Systems)	Synthetic	Low (when uninduced)	High if tightly regulated	Minimal background reduces silencing risk	Use only when needed; low basal expression is protective
Nos	Native bacterial	Moderate to high	Often unstable	Prone to methylation in plant genomes	Avoid as sole promoter in multi-gene constructs
Heat shock promoters	Native/engineered	Low	Stable when induced intermittently	Low basal expression limits silencing	Ideal for transient induction; combine with enhancers if needed

To counteract silencing and ensure durable transgene expression, several strategies have been developed. Using endogenous or species-specific promoters, such as ubiquitin or EF1α, can reduce the perception of the transgene as foreign and lower the risk of methylation-based silencing (Axelos et al., 1989; Christensen and Quail, 1996; Sivamani and Qu, 2006; Yoo et al., 2007). Another important factor is transgene copy number, low copy number or single-copy insertion events are less prone to both TGS and PTGS (Butaye et al., 2004)]. Avoiding repetitive sequences, such as tandem enhancer elements from CaMV35S, also helps reduce methylation susceptibility (Matzke and Matzke, 2004; Tang et al., 2007).The use of genomic insulators or S/MARs has been shown to protect transgene expression from positional effects and chromatin silencing by maintaining an open chromatin structure (Allen et al., 2000; Kurbidaeva and Purugganan, 2021). Furthermore, inducible or tissue-specific promoters can reduce the overall transcriptional load on host cells and minimize unintended triggering of PTGS (Desfeux et al., 2000; Feng et al., 2022). Finally, generating and screening multiple independent transgenic lines remains a best practice to identify those with stable and robust expression profiles suitable for long-term or commercial applications.

## 8 Challenges and future perspectives

Despite remarkable progress in promoter engineering in plant molecular farming, several technical and practical challenges continue to limit the full potential of recombinant protein production in plant systems (Liu and Stewart, 2016; Yasmeen et al., 2023). These challenges arise from the complexity of plant regulatory networks, the variability in transgene expression, and the limited predictability of promoter performance across different species, tissues, and production platforms (Peremarti et al., 2010; Gao et al., 2025). Addressing these issues will require an interdisciplinary approach combining synthetic biology, genomics, computational modeling, and advanced molecular tools.

### 8.1 Variability and context dependency

While previous sections (Sections 6 and 7) detail how promoter performance is shaped by species-, platform-, and epigenetic-context dependencies, such variability remains a major translational bottleneck, limiting the predictability and transferability of expression outcomes from laboratory-scale studies to industrial applications.

At the mechanistic level, promoter performance is shaped by chromatin context, including DNA methylation, histone modifications, and genomic integration site effects, which can cause substantial expression differences among independent transgenic events even when identical constructs are used (Meyer, 2015; Rajeevkumar et al., 2015; Magembe et al., 2023). Epigenetic silencing further contributes to instability, particularly under conditions of high transcriptional load or prolonged expression, such that increasing promoter strength can paradoxically reduce long-term expression robustness (Butaye et al., 2004; Matzke and Matzke, 2004; Tang et al., 2007).

In addition, promoter activity is modulated by developmental, physiological, and environmental factors, including tissue differentiation, growth conditions, and culture age. Together, these influences underscore that promoter behavior cannot be fully decoupled from genomic and cellular context and that achieving reproducible expression will require integrated strategies combining promoter design with construct architecture, transformation approaches, and process optimization.

### 8.2 Limited availability of well-characterized promoters

Although numerous constitutive, inducible, and tissue-specific promoters have been reported, only a small subset has been thoroughly characterized in terms of strength, dynamics, and expression consistency (Nayak et al., 2025). Moreover, there is a lack of standardized, modular promoter parts that can be easily assembled, tested, and reused across different systems (Cai et al., 2020). This hampers rational design and often leads to trial-and-error approaches in promoter selection. In addition, inducible promoters can suffer from leaky expression, high background activity, or toxicity of chemical inducers, especially at commercial scales (Zuo and Chua, 2000). Many tissue-specific promoters are relatively weak and may not drive sufficient expression for industrial needs without further engineering (Peremarti et al., 2010; Ali and Kim, 2019).

### 8.3 Regulatory and biosafety considerations

From a regulatory standpoint, the use of certain promoter elements, particularly those derived from plant viruses (e.g., CaMV35S), can raise concerns about horizontal gene transfer, transgene escape, and public perception (Tschofen et al., 2016). There is increasing demand for biosafe, non-viral, or synthetic promoters that are functionally robust yet regulatory-friendly, especially for pharmaceutical applications or food crops (Dey et al., 2015; Szarzanowicz et al., 2024). Developing synthetic promoters that are functionally decoupled from native plant sequences, yet maintaining high activity and specificity, is an important step toward regulatory compliance and public acceptance. However, this requires better understanding of cis-regulatory logic and synthetic enhancer design.

### 8.4 Complexity of multigene expression

Many recombinant protein production strategies, especially those involving multimeric proteins, metabolic pathways, or modular protein assemblies, require coordinated expression of multiple genes (Daniell and Dhingra, 2002). Engineering promoters that allow fine-tuned, balanced expression of several transgenes simultaneously remain a key challenge. Achieving stoichiometric expression of multiple subunits is particularly critical for assembling functional protein complexes or optimizing flux through engineered biosynthetic pathways (Lopez-Arredondo and Herrera-Estrella, 2012; Rajput et al., 2025). While polycistronic constructs, bidirectional promoters, and synthetic operon-like systems have been explored, their regulatory behavior in plant systems is often unpredictable due to transcriptional interference, promoter competition, or position effects (Krichevsky et al., 2012; Rajput et al., 2025). Therefore, precise promoter engineering and orthogonal regulatory systems remain essential for achieving coordinated multigene expression in plant molecular farming.

### 8.5 Future perspectives

**Figure 5:**
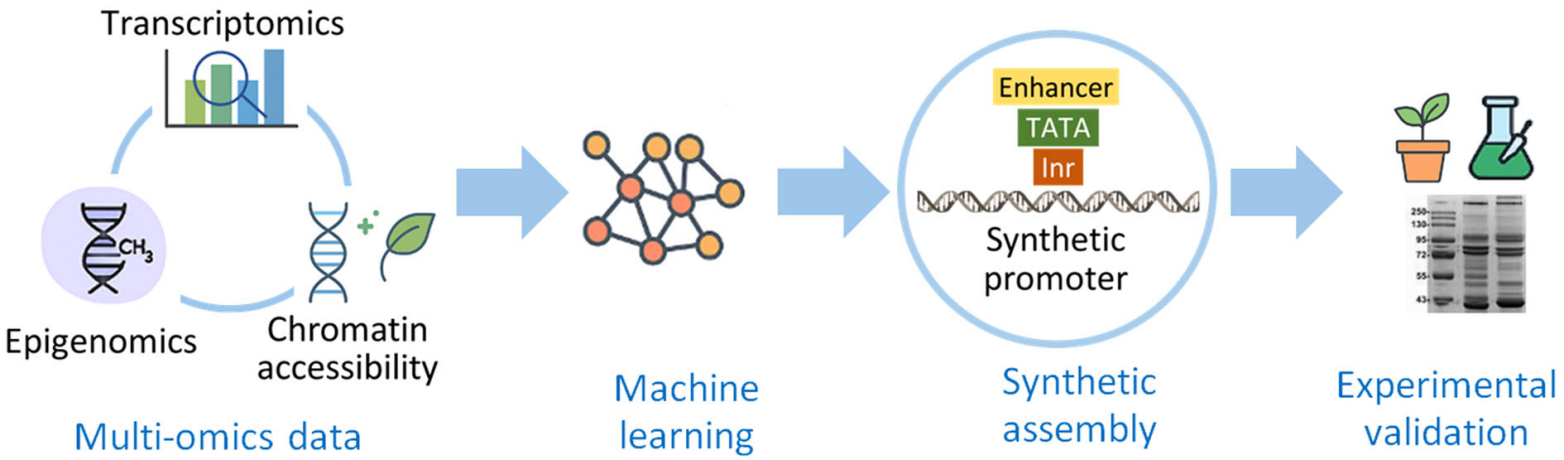
Data-driven workflow for rational promoter design. Multi-omics data, including transcriptomic (gene expression strength and tissue specificity), epigenomic (DNA methylation and histone modification patterns), and chromatin accessibility profiles (maps of open promoter regions), feed into computational models that guide the rational design and experimental validation of synthetic promoters with predictable transcriptional activity.

Addressing current barriers in plant molecular farming will require a shift toward predictive, data-driven frameworks and integrative design strategies. Future progress will be driven by the convergence of promoter engineering with multi-omics analyses, computational modeling, and synthetic circuit design. Integrating transcriptomic, epigenomic, and chromatin accessibility datasets provides a powerful foundation for deciphering promoter architecture and regulatory function (**Figure 5****).** High-resolution omics analyses can uncover transcription factor networks, active enhancer regions, and chromatin states linked to strong or context-specific expressions. By systematically correlating promoter sequence features with chromatin accessibility and gene expression profiles, it becomes possible to rationally design synthetic promoters with improved precision, robustness, and cross-species portability.

The following strategic research directions are particularly promising. These advances position promoter engineering as a dynamic and enabling technology for next-generation plant molecular farming.

- High-throughput screening and characterization: The development of scalable platforms to systematically evaluate large libraries of natural and synthetic promoters across species, tissues, and production contexts will accelerate the identification of robust regulatory elements and reduce reliance on empirically favored but context-limited promoters.
- Machine learning and computational modeling: Integrating deep learning with promoter sequence datasets can help predict promoter strength, tissue specificity, and inducibility. Tools like PlantProm DB (Shahmuradov et al., 2003) and PlantRegMap (Tian et al., 2020) can enable such predictions, but next-generation models incorporating epigenetic and chromatin features will be essential for improving predictive accuracy.
- Synthetic regulatory networks: Inspired by advances in microbial and mammalian systems, plant synthetic biology is beginning to employ logic gates, toggle switches, feedback loops, and biosensors to enable programmable, fine-tuned control of gene expression (Khan and Lister, 2025; Lloyd et al., 2025). Promoters designed to work within these systems will unlock more sophisticated applications.
- CRISPR-based gene regulation: dCas9-based activators and repressors targeted to synthetic promoter regions provide highly specific, tunable, and reversible control of gene expression without altering genomic DNA (Gondalia et al., 2025). This approach adds a modular regulatory layer that can complement traditional promoter engineering.
- Biosafety-by-design: Next-generation synthetic promoters should be both effective and biosafe, incorporating minimal homology with endogenous plant sequences, spatially confined expression domains, and built-in fail-safe mechanisms to prevent unintended gene flow or persistence.

## 9 Conclusion

Promoter engineering has become a critical tool for optimizing recombinant protein production in plant systems for molecular farming, which are increasingly recognized for their scalability, biosafety, and cost-effectiveness. However, the evidence synthesized in this review clearly demonstrates that effective promoter deployment must move beyond promoter strength as the primary design criterion. Instead, promoter performance is strongly influenced by genomic context, species-specific regulatory landscapes, transformation platforms, and epigenetic stability, factors that collectively determine reproducibility, yield consistency, and translational reliability. These findings underscore the need for rational, platform- and species-specific promoter engineering strategies that explicitly integrate biological constraints with production objectives. Prioritizing robustness, stability, and regulatory predictability over maximal transcriptional output is essential for advancing scalable and compliant plant-based biomanufacturing.

Moving forward, the integration of promoter engineering with synthetic biology, computational design, and high-throughput screening is expected to yield highly tunable, orthogonal, and context-resilient promoters. Emerging tools like CRISPR-based transcriptional regulation and modular synthetic circuits will further expand the functional versatility of engineered promoters. As these technologies continue to evolve and are validated beyond model systems, promoter engineering will remain central to advancing plant molecular farming and unlocking the full potential of plants as biofactories for high-value therapeutic and industrial proteins and enzymes.
